# Methylene blue therapy in addition to standard treatment for acute-phase septic shock: a pilot randomized controlled trial

**DOI:** 10.3389/fmed.2024.1431321

**Published:** 2024-10-14

**Authors:** Fabio Luis-Silva, Mayra Gonçalves Menegueti, Leandro Moreira Peres, Corina dos Reis Sepeda, Maria Cecília Jordani, Fabiola Mestriner, Bruno Cesar Petroski-Moraes, Joaquim Pedro Brito-de-Sousa, Ismael Artur Costa-Rocha, Bruna Lemos Cruz, Mariana Dermínio Donadel, Felipe Barizza de Souza, Gustavo Henrique Martins Reis, Fernando Bellissimo-Rodrigues, Anibal Basile-Filho, Christiane Becari, Paulo Roberto Barbosa Evora, Olindo Assis Martins-Filho, Maria Auxiliadora-Martins

**Affiliations:** ^1^Division of Intensive Care Medicine, Department of Surgery and Anatomy, Ribeirão Preto Medical School, University of São Paulo, São Paulo, Brazil; ^2^Ribeirão Preto Nursing School, University of São Paulo, São Paulo, Brazil; ^3^Division of Cardiac Surgery, Department of Surgery and Anatomy, Ribeirão Preto Medical School, University of São Paulo, São Paulo, Brazil; ^4^Division of Vascular and Endovascular Surgery, Department of Surgery and Anatomy, Ribeirão Preto Medical School, University of São Paulo, São Paulo, Brazil; ^5^René Rachou Institute, Oswaldo Cruz Foundation, FIOCRUZ-Minas, Belo Horizonte, Brazil; ^6^Department of Social Medicine, Ribeirão Preto Medical School, University of São Paulo, São Paulo, Brazil; ^7^Department of Surgery and Anatomy, Ribeirão Preto Medical School, University of São Paulo, Ribeirão Preto, Brazil; ^8^Department of Biological Sciences, Bauru School of Dentistry, University of São Paulo, Bauru, São Paulo, Brazil

**Keywords:** methylene blue, septic shock, lactate, nitric oxide, cytokines, vasopressors, norepinephrine, vasopressin

## Abstract

**Purpose:**

Methylene blue (MB) has been used to increase blood pressure in patients with septic shock by acting on guanylate cyclase and nitric oxide synthase.

**Objective:**

To determine whether the administration of MB to patients in the initial phase of septic shock leads to a reduction in the use of vasopressors compared to the Control group.

**Methods:**

This was a 1:1 randomized clinical trial of two groups (MB and Control). Forty-two patients were included in the present study; 23 patients were allocated to the Control group, and 19 were randomized to the MB group. Both groups had access to standard treatment, consisting of fluid replacement, vasopressors, and antibiotic therapy. Patients received a loading dose of MB (3 mg/kg) and maintenance (0.5 mg/kg/h) for 48 h. Vasopressor doses, laboratory test results, inflammatory and anti-inflammatory cytokine levels, and hemodynamic monitoring were recorded before the infusion of MB (T1) and after 20 min (T2), 2 h (T3), 24 h (T4), 48 h after the infusion started (T5) and 24 h after weaning (T6).

**Results:**

MB therapy was started together with the indication of vasopressin (VAS) as a second vasopressor. The MB group showed an immediate reduction in NOR dosage, an earlier reduction in VAS dosage, and higher IL-10 levels compared to the Control group.

**Conclusion:**

Early administration of MB in combination with standard treatment for septic shock might be reduce vasopressors dose. Continuous infusion of MB for 48 h was considered safe and there was no adverse events. These results highlight the potential of MB as a safe adjuvant therapeutic option in the treatment of septic shock.

**Clinical trial registration:**

https://clinicaltrials.gov/, identifier RBR-96584w4.

## Introduction

Septic shock, characterized by hemodynamic changes and systemic inflammation, is a leading cause of morbidity and mortality in intensive care units (ICU) (1, 2). The mortality rate varies from 38 to 46.5% (3), which represents a significant challenge for global health, especially in developing countries (4).

A diagnosis of septic shock occurs in patients with a confirmed or presumed focus of infection associated with a mean arterial pressure (MAP) ≤ 65 mmHg and a lactate concentration ≥ 2.0 mmol/L after adequate volume replacement (1).

Standard treatment includes fluid replacement, vasopressors, and antibiotics, with low-dose corticosteroids in refractory cases (1, 5). However, high mortality rates persist, especially in developing countries. It is extremely important to study new medications that help maintain hemodynamic stability until the antibiotic acts and combats the infectious focus (6, 7).

Methylene blue (MB), a nonselective inhibitor of soluble guanylate cyclase (SGC) and nitric oxide synthase (NOS), is a heterocyclic aromatic compound from the phenothiazine class that has been used since the 19th century and has had proven hemodynamic effects since 1976 (2); additionally, MB has been used safely, with few side effects when it is used at adequate doses. The safety of intravenous administration and dosages of 1–3 mg/kg have been previously reported, and the authors state that excessive doses of MB result in adverse effects on visceral tissue perfusion. Doses greater than 40 mg/kg are lethal (3, 4).

The study by López et al. (5) demonstrated increased 28-day mortality with the use of the nitric oxide synthase inhibitor 546c88, most of the events responsible for the disparity between groups were associated with the cardiovascular system (e.g., decreased output cardiac, pulmonary hypertension, systemic arterial hypertension, and heart failure). Later Khanna et al. (7) also demonstrated concern about the use of the nitric oxide synthase inhibitor 546c88 in patients with low cardiac output. However, these findings have not been reported specifically with the use of MB, on the contrary, Sari-Yavuz et al. (6) demonstrated the benefit of MB in patients with cardiogenic shock.

Several studies have proposed that MB can treat circulatory shock secondary to vasoplegia via nitric oxide (NO) (2, 8–14). Although the medical literature does not include robust studies on the use of MB in septic shock, even with a small sample, the results are promising regarding the association of MB with conventional treatment in these patients (8, 9, 11). In theory, the inhibition of excessive NO could act favorably, preventing systemic vasodilation and reducing microvascular injury in septic individuals. Another theory is that MB improves mitochondrial function and adenosine triphosphate (ATP) production in cells, helping to reverse cardiovascular dysfunction associated with septic shock (15).

Studies have shown the beneficial effects of MB in patients with septic shock (11, 16, 17); however, studies involving patients in the acute phase are rare (8, 9, 11).

A recently published meta-analysis compared the use of MB with control and showed that treatment with MB accelerated vasopressor discontinuation, reduced the time on mechanical ventilation and length of stay in the ICU unit (18).

Our hypothesis is that MB contributes to reducing the infusion of vasopressors, improving tissue perfusion and delaying mitochondrial death induced by nitric oxide if administered early in septic shock. Therefore, the aim of the present study was to determine whether the administration of MB to patients in the initial phase of septic shock leads to a reduction in the use of vasopressors, when used in combination with the standard treatment, compared to Control group, submitted to the standard treatment alone.

## Materials and methods

### Study design, population, and sampling

This was a pilot randomized clinical trial conducted in the ICU of a tertiary-care university hospital from January 2019 to August 2023. Simple randomization was performed using a computer-generated randomization list and used to prepare sealed, sequentially numbered envelopes. This list was administered by a researcher who did not know the patients nor actively participated in data collection. All the legal guardians of the included patients agreed and signed the free and informed consent form. The Institutional Review Board of the Hospital das Clínicas of the Ribeirão Preto Medical School approved this protocol–number: 562/2017, version: 2/2016.

The inclusion criteria were age > 18 years, septic shock diagnosis, use of norepinephrine/Vasopressin. A diagnosis of septic shock occurs in patients with a confirmed or presumed focus of infection associated with a mean arterial pressure (MAP) ≤ 65 mmHg and a lactate concentration ≥ 2.0 mmol/L after adequate volume replacement (1). The exclusion criteria were pregnancy, use of serotonergic agent, CD4 < 200/mm^3^, Neutrophils <500/mm^3^, patients in palliative care or at risk of imminent death. Withdrawals from the study occurred due to other causes of circulatory shock, *n* = 38. Blinding was not possible since MB leaves body fluids with a bluish-green color that are easily identified upon use. To minimize possible biases, the professional responsible for randomization had no access to patient clinical records, and the researcher did not know the patient group when performing hemodynamic monitoring for data collection.

**Figure 1:**
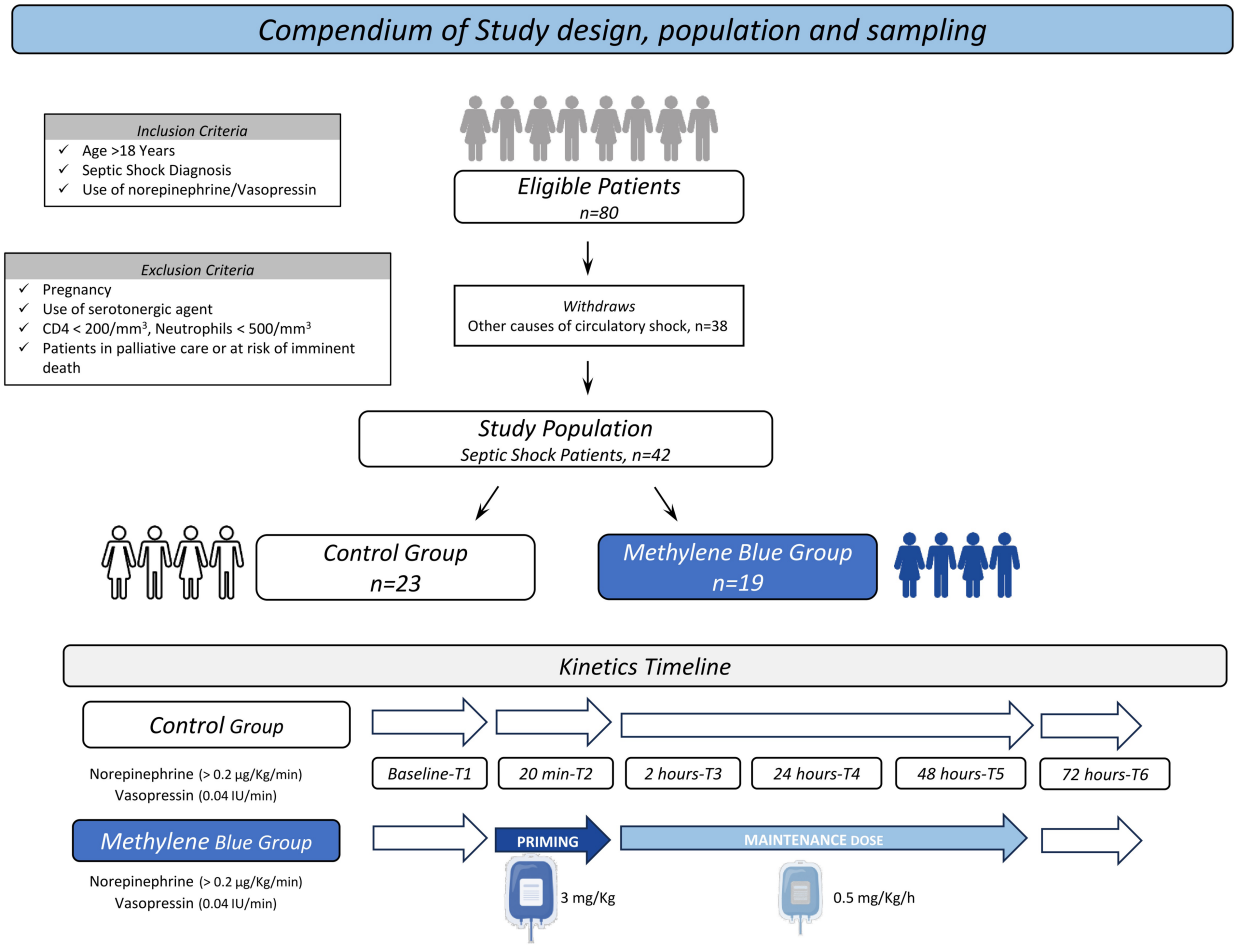
Compendium of study population and design. The flowchart illustrates the enrollment and randomization process for the selection of the study population.

Figure 1 summarizes the study population and study design. Eighty patients were enrolled upon signing the informed consent form by their next of kin and were randomly allocated into two groups according to the previously published protocol (12). After withdrawing based on clinical status (*n* = 38), a total of 42 patients were included in the present study; 23 patients were allocated to the Control group, and 19 were randomized to the MB group.

According to Figure 1, MB therapy was started together with the indication of vasopressin (VAS) as a second vasopressor, at this time the patient was using ≥0.2 μg/kg/min of norepinephrine and associated vasopressin at a dose of 0.04 IU/min.

No previous studies in the literature have used continuous-dose MB for 48 h to support the sample size calculation. Therefore, in the present investigation, the sample size was defined according to the guidelines proposed by Weingartner et al. (19) for pilot clinical trials, which recommended the inclusion of 20 patients in each study arm. After measuring and collecting initial exams, patients in the MB group received conventional treatment and MB at a dose of 3 mg/kg in 20 min and then 0.5 mg/kg/h for 48 h, and patients in the Control group received conventional treatment. Conventional treatment included fluid replacement, vasopressor drugs (noradrenaline and vasopressin), hydrocortisone (200 mg/day) and antibiotics within the first hour. After completion of the protocol, patients were followed up for 30 days to assess the outcome, discharge status, or death status. After randomization, the following procedures were performed: weight measurement using a bed scale (Stryker^®^ brand), catheter insertion in the femoral artery (VolumeView System^®^), and central venous access in the internal jugular or subclavian vein (Presep^®^) for monitoring with the EV1000^®^ platform (Edwards Lifesciences Corporation^®^) (20). The SAPS3 (Simplified Acute Physiology Score 3) and APACHE II (Acute Physiology and Chronic Health Evaluation) prognostic scores were calculated for the first 24 h after admission, and the daily SOFA score was calculated for the 4 days of the protocol.

### Hemodynamic and vasopressor drug monitoring

After calibrating the EV1000^®^ platform, the mean arterial pressure (MAP), cardiac output (CO), cardiac index (CI), heart rate (HR), systemic vascular resistance index (SVRI), central venous pressure (CVP), stroke volume index (SVI), global ejection fraction (GEF), pulmonary vascular permeability index (PVPI), global end diastolic volume index (GEDVI) and extravascular lung water index (ELWI) were measured. Measurements of central venous oxygen saturation (ScVO_2_), arterial blood gas, serum lactate, bilirubin, complete blood count, sodium, potassium, urea, and creatinine were also performed. The oxygen delivery index (DO_2_I), oxygen consumption index (VO_2_I), and oxygen extraction rate (O_2_ER) were calculated. Vasopressor drug monitoring (norepinephrine/NOR and vasopressin/VAS) was carried out daily by a physician according to a standardized institutional protocol.

### Laboratory analysis of immunological features

To evaluate the immunological parameters, serial whole-blood sampling was performed along the study timeline (T1–T6) using EDTA and heparin. Samples were subjected to centrifugation at 3,500 rpm for 10 min at 16°C, and plasma aliquots were stored at −80°C until processing. Soluble immune mediator [interleukin 8 (IL-8/CXCL8), interleukin 6 (IL-6), tumor necrosis factor alpha (TNF-α), and interleukin 10 (IL-10)] levels were measured in EDTA-treated samples according to the manufacturer’s instructions (R&D Systems). The results of soluble immune mediators are expressed in pg./mL. The nitric oxide (NO_3_) levels were measured in heparin-treated samples via chemiluminescence according to Dweik et al. (21). The results on NO are expressed in μM.

### Statistical analysis

The chi-square test was used to verify the associations of qualitative variables between the MB and Control groups. Student’s *t*-test was used to analyze quantitative clinical variables. The comparison of immunological features between the MB and Control groups at each timepoint was performed by the Mann–Whitney test. The Spearman rank test was used to perform cross-correlation analysis of vasopressor drugs and hemodynamic and immunological variables (attributes) among all timepoints. GraphPad Prism software was used for all the statistical analyses. In all cases, *p* < 0.05 was considered to indicate statistical significance. Significant correlations were used to construct integrative networks. Integrative networks were built using the systems biology approach of the Cytoscape open-source platform (available at https://cytoscape.org) based on the “r” scores of significant correlations. The networks were assembled using a cluster layout with nodes used to represent each variable, and connecting lines were employed to identify positive (continuous line) and negative (dashed line) correlations. The node sizes are proportional to the number of correlations between parameters. Line thickness illustrates the correlation strength, ranging from weak/moderate (“r” scores from |0.1 to 0.6|, thin lines) to strong correlations (“r” scores from ≥ |0.7|, thick lines). The red line illustrates the correlations between immunological features and vasopressor drugs. Correlation matrices were assembled using the “corrplot” package of R software (Project for Statistical Computing Version 3.0.1). Microsoft Excel and Prism GraphPad software were used to create the graphics.

## Results

The sample comprised forty-two patients. Table 1 summarizes the demographic and clinical characteristics of the study population.

**Table 1:** Demographic and clinical features of the study population.

Variables	Group		
	Control (*n* = 23)	MB (*n* = 19)	*p*-value*
Sex (male)—n (%)	16 (70)	14 (74)	0.77
Age (years)—Mean	42.7 ± 17.7	51.5 ± 14.3	0.09
Hypertension—n (%)	7 (30)	6 (32)	0.93
Diabetes—n (%)	4 (17)	3 (16)	0.89
Acute kidney injury—n (%)	14 (61)	15 (79)	0.21
Hemodialysis—(%)	6 (27)	2 (11)	0.20
SAPS3—Mean ± SD	58.2 ± 9.8	78.1 ± 11.4	*<0.0001*
Death Risk—Mean ± SD	47.1 ± 20.6	82.0 ± 13.5	*<0.0001*
APACHE II—Mean ± SD	21.7 ± 9.6	39.6 ± 6.8	*<0.0001*
SOFA—Mean ± SD			
T1 (Baseline)	9.0 ± 2.7	12.1 ± 2.8	*0.001*
T4 (24 h)	8.2 ± 3.1	11.2 ± 3.2	*0.004*
T5 (48 h)	7.3 ± 3.3	10.8 ± 3.4	*0.002*
T6 (72 h)	7.2 ± 3.7	10.5 ± 3.4	*0.004*
Sites of infection			
Abdomen—n (%)	5 (21.7)	7 (36.8)	0.30
Lung—n (%)	15 (65.1)	6 (31.6)	*0.03*
Urinary tract—n (%)	1 (4.4)	3 (15.8)	0.25
CRBSI—n (%)	1 (4.4)	0 (0)	0.33
Other—n (%)	1 (4.4)	3 (15.8)	0.25
Positive culture—n (%)	13 (56.5)	13 (68.4)	0.44
Effectiveness of empiric antibiotic—(%)	43.5%	21.1%	0.12
Days of antibiotic use—Mean ± SD	18.9 ± 20.0	16.4 ± 12.5	0.64
Shock time at baseline (hour)—Mean ± SD	44.5 ± 24.5	33.3 ± 12.3	0.07
Fluid balance (mL in 72 h)—Mean ± SD	1,416 ± 3,158	2,871 ± 3,163	0.15
30 days outcome (Death)—n (%)	14 (61)	9 (47)	0.38

* MB, Methylene blue; SD, standard deviation; CRBSI, Catheter-Related Blood Stream Infection. Chi-square test was used for categorical data and Student’s *t*-test employed for continuous variables comparisons. Significant differences are underscored in italic.

### MAP and vasopressor doses along the kinetic timeline in the MB and control groups

**Figure 2:**
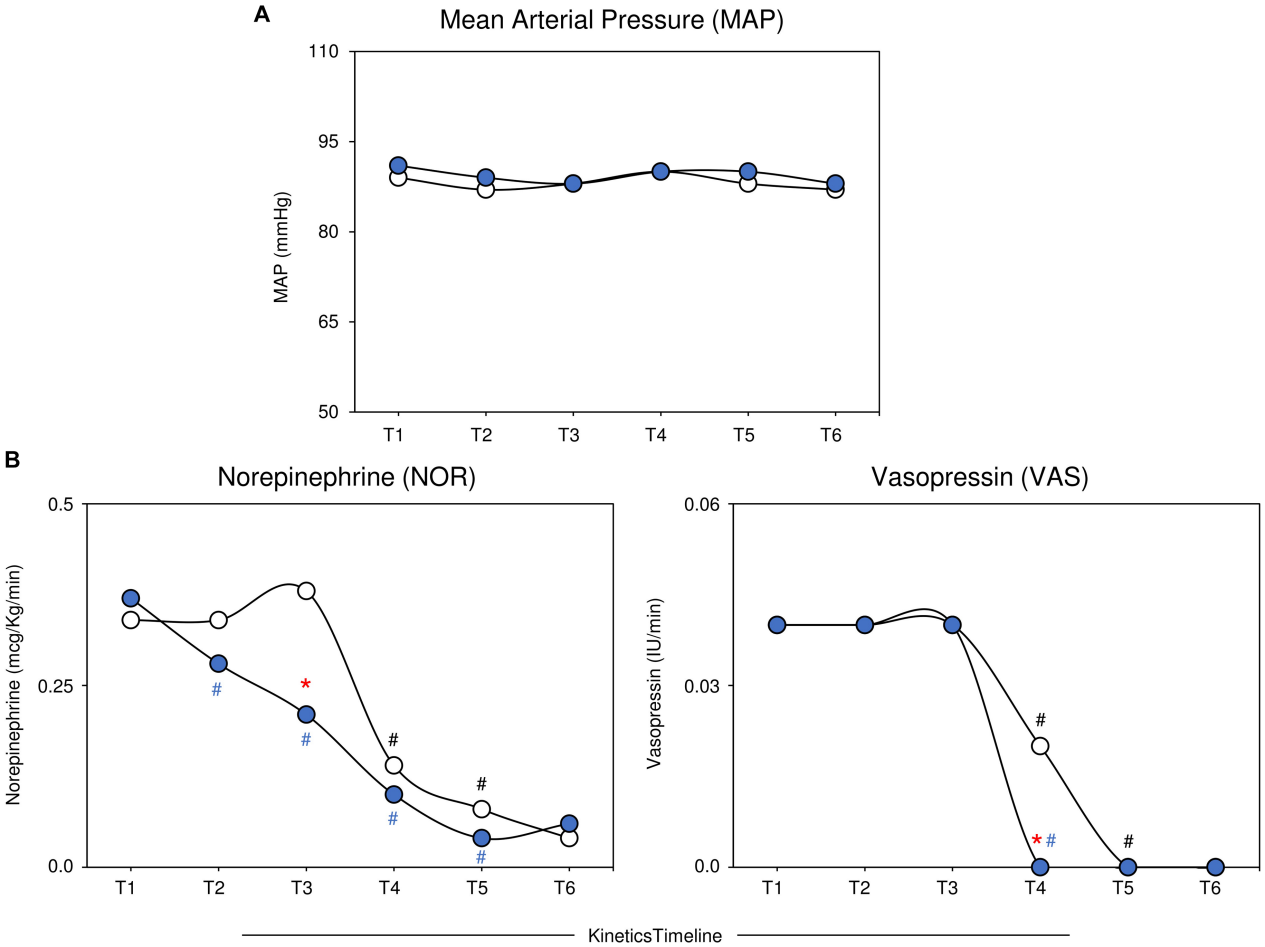
Profile of MAP and vasopressor doses (NOR and VAS) along the kinetics timeline in the MB and Control groups. The data are reported as medians for the MB (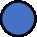, *n* = 19) and control (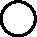, *n* = 23) groups. The analysis of MAP and vasopressor doses along the kinetic timeline (T1–T6) was assessed by comparing pairs of adjacent timepoints via the Mann–Whitney test, and *p* < 0.05 was considered to indicate statistical significance. Intragroup significant differences were underscored by (**#** and **#** for the MB and Control groups, respectively). A comparative analysis between the MB and Control groups at the matching timepoint was carried out by the Mann–Whitney test, and *p* < 0.05 was considered to indicate statistical significance. Significant differences between the MB and Control groups at matching timepoints are highlighted by *. MB, methylene blue; MAP, mean arterial pressure; NOR, norepinephrine; VAS, vasopressin.

The analysis of MAP and vasopressor doses along the kinetic timeline was assessed by comparing pairs of adjacent timepoints. Figure 2 shows the median MAP values along with the NOR and VAS scores along the kinetic timelines in the MB and Control groups. The data analysis did not reveal significant differences in the MAP during the kinetics follow-up. The NOR dose was markedly lower in the MB test at T2 than at T1, with a continuous decrease toward T5, while in the Control group, a decrease in NOR was observed later at T4 than at T3. A significant difference in NOR dose between the MB and Control groups was observed at T3. The analysis of VAS scores demonstrated early withdrawal in the MB group at T4, while in the Control group, VAS scores were withdrawn later at T5. A significant difference in VAS score between the MB and Control groups was observed at T4 (Figure 2). Analysis of several hemodynamic variables (CO, CI, HR, SVRI, CVP, SVI, GEF, PVPI, GEDVI and ELWI) along the kinetics timeline (T1 to T6) did not reveal significant differences (Supplementary Table S1). A detailed description of the median values of MAP and vasopressor doses along the kinetic timeline is provided in Supplementary Table S2.

Invasive hemodynamic monitoring was important to demonstrate the absence of persistent hypovolemia or cardiac dysfunction as explanations for circulatory shock, demonstrating the real presence of septic shock in both groups (MB and Control).

### Kinetic timeline of serum lactate concentration, DO_2_I, VO_2_I, and O_2_ER × CO in the MB and control groups

**Figure 3:**
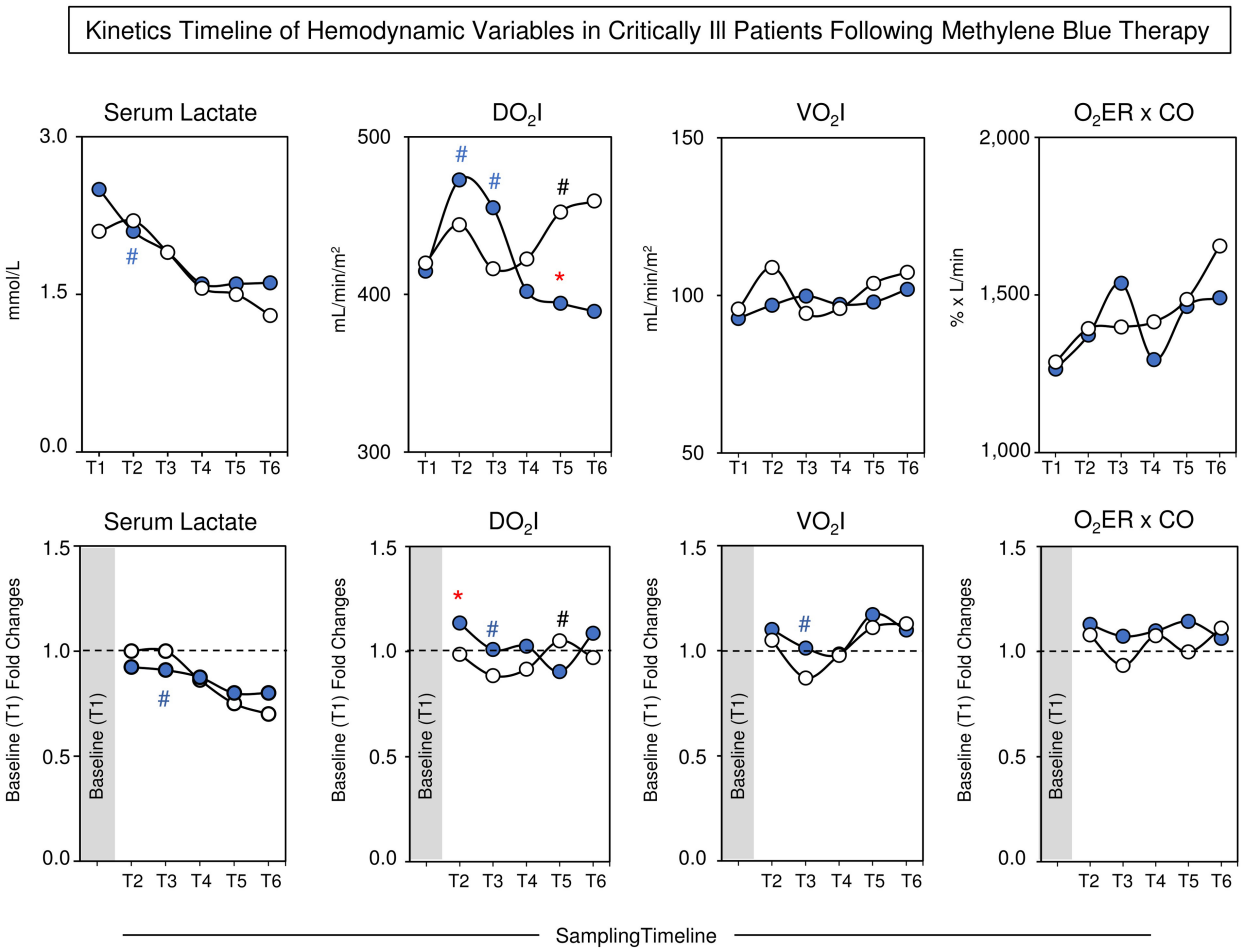
Kinetics timeline of hemodynamic variables (serum lactate concentration, DO_2_I, VO_2_I, and O_2_ER × CO) along the kinetics timeline in the MB and Control groups. The data are reported as median values (upper panels) and baseline (T1) fold changes (lower panels) for the MB (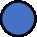, *n* = 19) and control (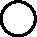, *n* = 23) groups. The analysis of hemodynamic variables along the kinetic timeline (T1–T6) was assessed by comparing pairs of adjacent timepoints via the Mann–Whitney test, and *p* < 0.05 was considered to indicate statistical significance. Intragroup significant differences were underscored by(**#** and **#** for the MB and Control groups, respectively). A comparative analysis between the MB and Control groups at the matching timepoint was carried out by the Mann–Whitney test, and *p* < 0.05 was considered to indicate statistical significance. Significant differences between the MB and Control groups at matching timepoints are highlighted by *. MB, methylene blue; DO_2_I, oxygen delivery index; VO_2_I, oxygen consumption index; O_2_ER, oxygen extraction rate; CO, cardiac output.

The analysis of hemodynamic variables (serum lactate concentration, DO_2_I, VO_2_I and O_2_ER × CO) was assessed along the kinetic timeline by comparing pairs of adjacent timepoints. Figure 3 shows the median values of the serum lactate concentration, DO_2_I, VO_2_I, and O_2_ER × CO. Data analysis demonstrated that while the serum lactate concentration decreased in the MB group at T2 compared to T1, no differences were observed between adjacent timepoints in the Control group. The analysis of DO_2_I demonstrated an early increase in MB at T2 and T3 and a later increase at T5 in the Control group, leading to differences between the MB and Control groups. No significant differences were observed for VO_2_I or O_2_ER × CO along the kinetics timeline. The analysis of fold changes further corroborated these findings (Figure 3). A detailed description of the median values of the serum lactate concentration, DO_2_I, VO_2_I and O_2_ER × CO concentration along the kinetic timeline is provided in Supplementary Table S2.

### Changes in plasma immune mediator and nitric oxide levels in the MB and control groups

**Figure 4:**
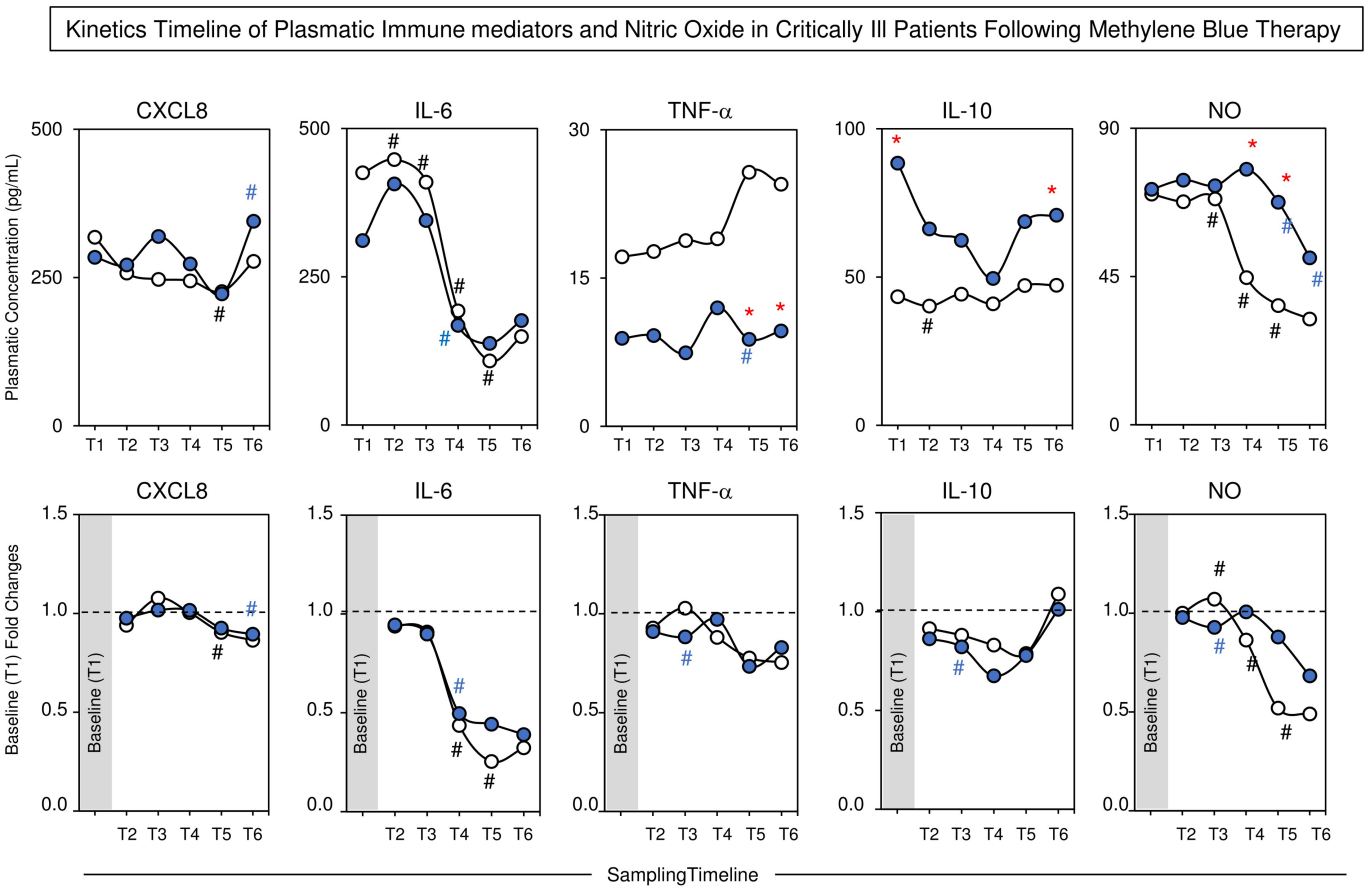
Kinetics timeline of plasmatic immunomediators (CXCL8, IL-6, TNF-α and IL-10) and nitric oxide (NO) during the kinetics timeline in the MB and Control groups. The data are reported as median values (upper panels) and baseline (T1) fold changes (lower panels) for the MB (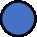, *n* = 19) and control (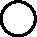, *n* = 23) groups. The analysis of plasmatic immunomediators and nitric oxide concentrations along the kinetic timeline (T1–T6) was performed by comparing pairs of adjacent timepoints via the Mann–Whitney test, and *p* < 0.05 was considered to indicate statistical significance. Intragroup significant differences were underscored by(**#** and **#** for the MB and Control groups, respectively). A comparative analysis between the MB and Control groups at the matching timepoint was carried out by the Mann–Whitney test, and *p* < 0.05 was considered to indicate statistical significance. Significant differences between the MB and Control groups at matching timepoints are highlighted by *. MB = methylene blue; CXCL8 = interleukin 8 (IL-8); IL-6 = interleukin 6; TNF-α = tumor necrosis factor alpha; IL-10 = interleukin 10.

Figure 4 presents the overall profile of soluble plasma immune mediators and NO in the MB and Control groups. These immune mediators were chosen because they are related to the inflammatory response in sepsis, thus making it possible to evaluate whether treatment with MB alters the inflammatory response. The analysis of immune mediators and NO was assessed along the kinetic timeline by comparing pairs of adjacent timepoints. The data analysis demonstrated an increase in CXCL8 in the MBs at T6 compared to T5 and a decrease in the Control group at T5 compared to T4. The data showed an increase in IL-6 in the Control group at T2, with a progressive decrease toward T5 and a decrease in MB at T4 compared to T3. Compared with those in the Control group, TNF-α in the MB at T5 were lower than those in T4, and lower levels were detected in the MB at T5 and T6. The analysis of IL-10 showed lower levels in the Control group at T2 than at T1. Higher levels of IL-10 were observed in the MBs at T1 and T6 than in the Control group. NO was elevated at T3 in the Control group and progressively decreased toward T5. In the MB group, the levels of NO were greater than those in the Control group at T4 and T5 and displayed a progressive decrease toward T6. The analysis of fold changes corroborated these findings. A detailed description of the median values of plasma immune mediators and nitric oxide concentrations along the kinetic timeline is provided in Supplementary Table S3.

### Integrative networks of vasopressor drugs and hemodynamic and immunological variables in the MB and control groups

**Figure 5:**
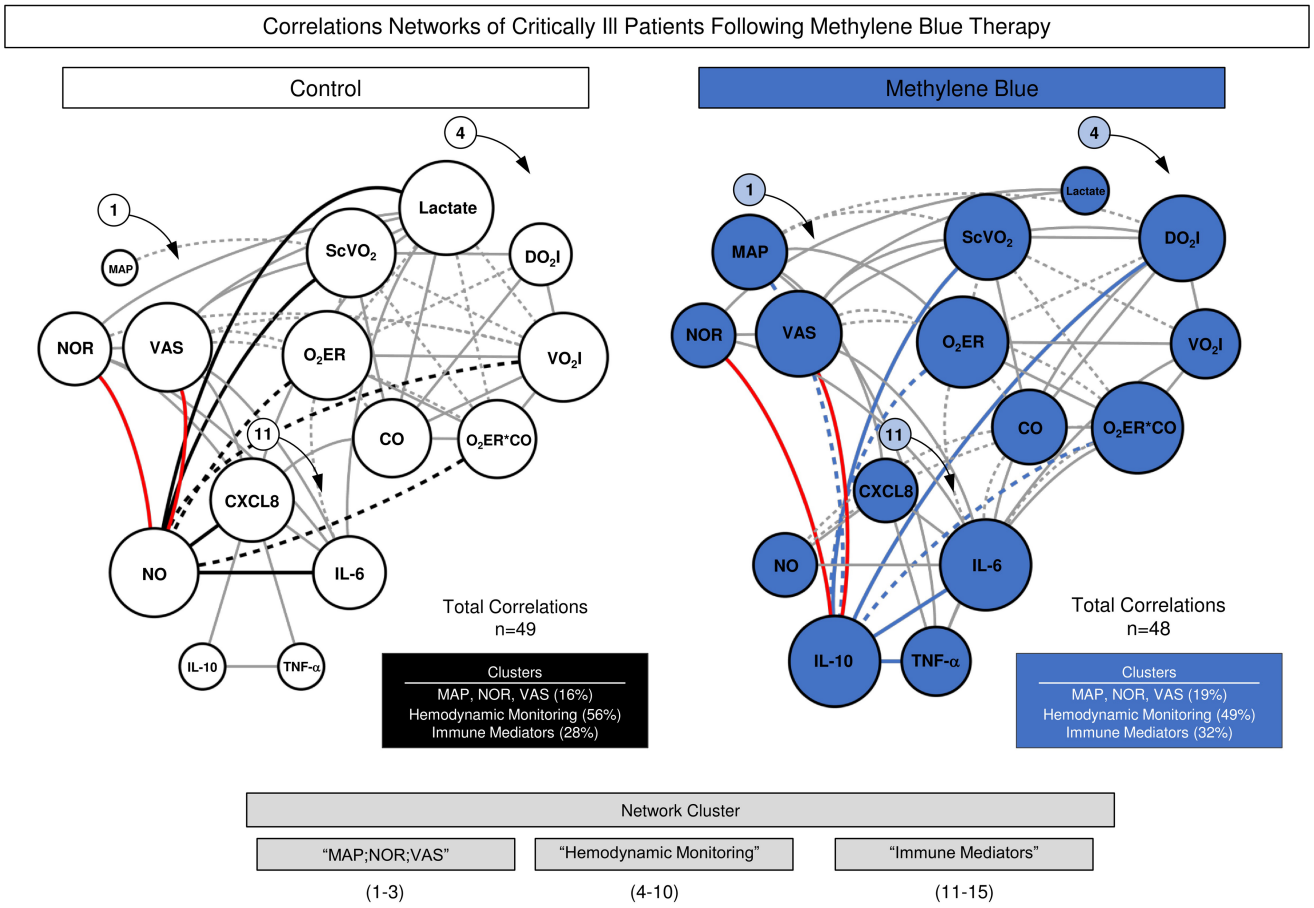
Integrative networks of vasopressor drugs and hemodynamic and immunological variables in the MB and Control groups. Integrative networks of vasopressor drugs and hemodynamic and immunological variables were assembled for the MB (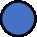) and control (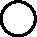) groups. The Spearman rank test was used to perform cross-correlation analysis among all timepoints. Integrative networks were built using systems biology approaches (Cytoscape open-source platform available at https://cytoscape.org) using a cluster layout with nodes used to represent each variable and connecting lines used to identify positive (continuous line) and negative (dashed line) correlations. The node sizes are proportional to the number of correlations between parameters. Line thickness illustrates the correlation strength, ranging from weak/moderate (“r” scores from |0.1 to 0.6|, thin lines) to strong correlations (“r” scores from ≥ |0.7|, thick lines). The red line illustrates the correlations between immunological features and vasopressor drugs.

Figure 5 displays the integrative network of vasopressor drugs and hemodynamic and immunological variables in the MB and Control groups. Integrative networks were built using systems biology approaches with a cluster layout with nodes used to represent each variable and connecting lines used to identify positive and negative correlations between pairs of attributes. The node sizes are proportional to the number of correlations. Despite the similar numbers of correlations observed in the MB and Control groups (*n* = 48 and *n* = 49, respectively), intracluster analysis demonstrated greater contributions of the “immune mediators” (32% vs. 28%) and “MAP;NOR;VAS” clusters in the MB (19% *vs* 16%) cohort than in the Control group. Conversely, more correlations within the “Hemodynamic Monitoring” (56% vs. 49%) cluster were observed in the Control group than in the MB group. Notably, while NOR and VAS scores were directly correlated with NO in the Control group, they were directly correlated with IL-10 in the MB group (Figure 5, red connecting lines). Overall, the integrative network analysis demonstrated that, while NO represents a key attribute orchestrating the correlation with the “Hemodynamic Monitoring” cluster in the Control group, IL-10 plays a pivotal role in coordinating the correlation with the “Hemodynamic Monitoring” cluster (Figure 5, thick connecting lines). This was more evident for the ScVO_2_, O_2_ER and O_2_ER × CO attributes. Taken together, these findings indicate that the underlying mechanism through which MBs impact hemodynamic features in septic shock may include not only the suppression of NO activity but also the involvement of other events mediated by IL-10. Detailed correlogram data supporting the integrative network are provided in Supplementary Figure S1.

## Discussion

The present study demonstrated that methylene blue (MB) therapy, when initiated together with vasopressin as a second vasopressor, led to an immediate reduction in norepinephrine dosage and an earlier reduction in vasopressin dosage compared to the control group. The MB group was weaned from vasopressin within 24 h, while the control group required 48 h. Continuous AM infusion for 48 h resulted in a decrease in the use of vasopressors by 2 h, maintained throughout the infusion period.

Levels of nitrate, a metabolite of NO, were higher in the MB group during the first 48 h. The MB group also showed an increase in CXCL8 from T2 to T3, potentially benefiting the acute phase of septic shock. TNF-α levels in the MB group showed less pronounced increases compared to the control group, despite patients in the MB group having more severe diseases according to prognostic indices.

Our findings are in line with previous studies that suggest that early use of MA may reduce the duration of vasopressor use in patients with septic shock. This supports the idea that MB should be used as adjuvant therapy in the early stages of septic shock rather than as rescue therapy (18). The sustained hemodynamic improvements observed in our study contrast with the findings of Preiser et al., who reported only transient improvements with a single dose of AM. This difference can be attributed to our continuous infusion approach.

Our results regarding cytokine levels differ from those reported by Memis et al., who found no changes in the serum levels of several cytokines during 6 h of AM use. We speculate that the prolonged use of AM in our study may have triggered more sustained effects on inflammatory and anti-inflammatory responses.

A strength of our study is the use of integrative network analysis, which revealed different correlation patterns between vasopressor drugs and hemodynamic and immunological variables in the MB and control groups. This analysis suggests that the effects of MB on hemodynamics in septic shock may depend not only on the blockade of NO action, but also on events mediated by IL-10.

Our study also provides information on the immunomodulatory effects of MB, showing its potential to balance inflammatory and anti-inflammatory responses. The reduction in IL-10 levels in the MB group suggests that MB can modulate IL-10 release with potential effects on immune balance.

Finally, we also observed that patients in the MB group had more severe disease according to the SOFA, SAPS3 and APACHE II scores. However, patients in the MB group used fewer days of vasopressors and had a mortality rate in 30 days of 47% versus 61% in the Control group. However, this study was not designed to assess mortality. MB group, despite being more severe, presented an earlier vasopressor dose reduction.

Furthermore, in those patients with positive cultures, empirical antibiotic therapy based on the antibiogram was initiated less than 3 h after the diagnosis of septic shock, providing adequate coverage in 43.5% of the Control group and 21.1% of the MB group (*p* < 0.1240). However, all patients received antibiotics within a maximum of 3 h after diagnosis.

It is important to recognize some limitations of this study. Firstly, this is a single-center study with the small sample size. Secondly, it is important to mention that the study was not blind, due to the properties of MB, which can leave participants’ skin and secretions with a bluish tint, facilitating the identification of those who received the treatment. We emphasize that only the patients and medical staff were not blind. However, all patients were sedated and were not exposed to the placebo effect. Furthermore, the medical team strictly followed the institutional protocol for adjusting vasopressor doses.

Furthermore, during the coronavirus disease (COVID-19) pandemic, the study team decided not to recruit patients with this diagnosis, due to unknown pathophysiological aspects and doubts regarding the action of MB in this disease. For this reason, this trial lasted longer than expected because the pandemic reduced the recruitment rate. Finally, it is necessary to highlight that MB was only started after the indication of invasive hemodynamic monitoring, which in turn was only indicated after the patient was using two vasopressors (NOR and VAS), as septic shock with a low dose of vasopressor, does not indicates the need for invasive hemodynamic monitoring.

Taken together, our findings corroborate the benefits of MB in reducing the need for vasopressors to control the hemodynamic variables in septic patients. The pros include its different mechanism of action compared to adrenergic agents, which reduces adverse events such as myocardial ischemia and decreased regional blood flow. MB is also easy to administer through continuous infusion, has a low cost, and is widely available. However, the cons include the lack of increase in cardiac output and oxygen delivery and the absence of a clear understanding of dosage and timing of administration (22).

## Conclusion

The administration of MB in combination with standard treatment resulted in an earlier might be reduce vasopressors dose in the MB group, compared to the Control group that received only conventional treatment. Continuous infusion of MB for 48 h was considered safe and there was no adverse events. Furthermore, it demonstrated a modulation of inflammatory and anti-inflammatory mediators and serum nitrate levels. These results highlight the potential of MB as a safe adjuvant therapeutic option in the treatment of septic shock. However, larger and longer studies are needed to confirm our findings.

## Data Availability

The original contributions presented in the study are included in the article/Supplementary material, further inquiries can be directed to the corresponding author.
