# Supplementary material for: Methylene blue therapy in addition to standard treatment for acute-phase septic shock: a pilot randomized controlled trial
Source: Front Med (Lausanne). 2024 Oct 14;11:1431321. doi: 10.3389/fmed.2024.1431321 (PMC11514138; doi:10.3389/fmed.2024.1431321)
Supplement: Supplementary file 3 [file Table_1.DOCX]

Supplementary Table 1. Hemodynamic parameters in MB and Controls

| Parameter |  | T1 | | |  | T2 | | |  | T3 | | |  | T4 | | |  | T5 | | |  | T6 | | |
| --- | --- | --- | --- | --- | --- | --- | --- | --- | --- | --- | --- | --- | --- | --- | --- | --- | --- | --- | --- | --- | --- | --- | --- | --- |
|  |  | CO | MB | p |  | CO | MB | p |  | CO | MB | p |  | CO | MB | p |  | CO | MB | p |  | CO | MB | p |
| CO* |  | 6.9  (5.4-8.6) | 6.3  (5.1-7.4) | 0.240 |  | 7.0  (5.0-9.2) | 7.2  (5.7-7.8) | 0.317 |  | 6.5  (5.6-8.6) | 6.6  (4.5-7.5) | 0.282 |  | 5.8  (4.5-8.9) | 5.8  (4.3-6.7) | 0.283 |  | 7.1  (6.2-8.1) | 6.0  (4.9-7.4) | 0.086 |  | 6.7  (5.8-9.1) | 6.2  (5.4-7.0) | 0.122 |
| CI |  | 3.7  (3.2-4.6) | 3.6  (3.0-3.8) | 0.319 |  | 4.2  (3.0.2-5) | 4.1  (3.6-4.4) | 0.792 |  | 3.7  (3.2-4.7) | 3.7  (3.2-4.1) | 0.522 |  | 3.5  (2.6-4.4) | 3.2  (2.8-4.2) | 0.608 |  | 3.9  (3.5-4.5) | 3.5  (3.1-3.8) | 0.070 |  | 3.8  (3.4-4.8) | 3.6  (3.1-4.1) | 0.222 |
| HR |  | 92  (73-113) | 93  (78-105) | 0.886 |  | 95  (75-113) | 98  (85-110) | 0.507 |  | 86  (74-106) | 94  (82-102) | 0.579 |  | 87  (73-105) | 82  (69-95) | 0.525 |  | 92  (81-114) | 85  (74-98) | 0.194 |  | 92  (80-105) | 81  (69-115) | 0.373 |
| SVRI |  | 1,676  (1,343-2,311) | 1,812  (1,434-2,239) | 0.786 |  | 1,570  (1,284-1,847) | 1,563  (1,295-2,112) | 0.950 |  | 1,663  (1,246-2,161) | 1,562  (1,210-2,063) | 0.876 |  | 1,760  (1,342-2,275) | 1,895  (1,472-2,268) | 0.809 |  | 1,576  (1,391-1,836) | 1,797  (1,379-2,137) | 0.398 |  | 1,509  (1,242-1,928) | 1,590  (1,283-1,963) | 0.656 |
| CVP |  | 13  (7-21) | 11  (7-17) | 0.308 |  | 11  (8-15) | 11  (8-15) | 0.757 |  | 12  (9-16) | 10  (7-14) | 0.668 |  | 12  (11-16) | 11  (7-17) | 0.619 |  | 13  (7-15) | 12  (8-18) | 0.552 |  | 11  (8-13) | 10  (7-12) | 0.449 |
| SVI |  | 40  (32-50) | 36  (31-48) | 0.462 |  | 41  (33-52) | 38  (30-52) | 0.456 |  | 43  (34-50) | 37  (28-53) | 0.253 |  | 38  (32-45) | 39  (34-52) | 0.666 |  | 41  (39-53) | 45  (26-51) | 0.633 |  | 42  (36-47) | 47  (34-51) | 0.941 |
| GEF |  | 28  (21-32) | 23  (19-29) | 0.304 |  | 26  (21-33) | 26  (20-29) | 0.524 |  | 26  (22-33) | 26  (20-31) | 0.476 |  | 27  (20-34) | 26  (19-33) | 0.792 |  | 30  (22-35) | 27  (23-32) | 0.417 |  | 29  (21-36) | 29  (23-35) | 0.872 |
| PVPI |  | 1.9  (1.7-2.2) | 2.0  (1.6-2.6) | 0.421 |  | 1.8  (1.6-2.1) | 2.1  (1.6-2.7) | 0.262 |  | 1.9  (1.6-2.2) | 2.2  (1.7-2.6) | 0.199 |  | 2.0  (1.5-2.2) | 2.0  (1.9-2.4) | 0.370 |  | 2.0  (1.6-2.3) | 2.0  (1.8-2.5) | 0.790 |  | 1.9  (1.6-2.1) | 2.1  (1.6-2.5) | 0.433 |
| GEDVI |  | 660  (488-816) | 626  (570-749) | 0.956 |  | 659  (490-773) | 620  (548-707) | 0.599 |  | 661  (478-757) | 582  (521-692) | 0.398 |  | 669  (480-742) | 623  (548-746) | 0.988 |  | 666  (467-737) | 641  (562-717) | 0.639 |  | 606  (500-722) | 565  (542-740) | 0.979 |
| ELWI |  | 7.1  (6.0-10.3) | 8.4  (7.1-10.4) | 0.300 |  | 7.4  (6.4-10.6) | 7.7  (7.1-9.7) | 0.428 |  | 7.5  (6.4-9.9) | 8.1  (7.0-9.8) | 0.592 |  | 8.1  (6.5-9.1) | 9.2  (6.7-9.8) | 0.377 |  | 7.8  (6.7-9.2) | 8.4  (6.4-11) | 0.569 |  | 7.7  (6.6-9.3) | 8.8  (6.5-10.6) | 0.407 |

*CO=cardiac output (L/min); CI=cardiac index (L/min/m^2^); HR=heart rate (bpm); SVRI=systemic vascular resistance index (dynes.sec/cm^-5^/m^2^); CVP=central venous pressure (mmHg); SVI=stroke volume index (mL/m^2^); GEF=global ejection fraction (%); PVPI=pulmonary vascular permeability index; GEDVI=global end diastolic volume index (mL/min); ELWI=extravascular lung water index (mL/kg). Data are reported as median values (interquartile range). Comparative analysis between Control x MB groups were assessed by Mann-Whitney test and significant differences considered at p<0.05.
