# Supplementary material for: Methylene blue therapy in addition to standard treatment for acute-phase septic shock: a pilot randomized controlled trial
Source: Front Med (Lausanne). 2024 Oct 14;11:1431321. doi: 10.3389/fmed.2024.1431321 (PMC11514138; doi:10.3389/fmed.2024.1431321)
Supplement: Supplementary file 4 [file Table_2.DOCX]

| Variables |  | Kinetics Timeline | | | | | | | | | | | | | | | | |
| --- | --- | --- | --- | --- | --- | --- | --- | --- | --- | --- | --- | --- | --- | --- | --- | --- | --- | --- |
|  |  | T1 | |  | T2 | |  | T3 | |  | T4 | |  | T5 | |  | T6 | |
|  |  | Control | MB |  | Control | MB |  | Control | MB |  | Control | MB |  | Control | MB |  | Control | MB |
| MAP |  | 89  (74-100) | 91  (69-111) |  | 87  (70-97) | 89  (66-103) |  | 88  (68-94) | 88  (68-113) |  | 90  (68-98) | 90  (70-116) |  | 88  (70-93) | 90  (70-114) |  | 87  (63-92) | 88  (44-105) |
| Norepinephrine |  | 0.34  (0.12-1.64) | 0.37  (0.2-1.5) |  | 0.34  (0.12-1.47) | 0.28  (0.1-1.0) |  | 0.38  (0.12-1.22) | **0.21***  **(0.0-0.5**) |  | 0.14  (0.0-1.0) | 0.1  (0.0-1.45) |  | 0.08  (0.0-1.22) | 0.04  (0.0-0.75) |  | 0.04  (0.0-2.0) | 0.06  (0.0-2.0) |
| Vasopressin |  | 0.04  (0.04-0.04) | 0.04  (0.04-0.04) |  | 0.04  (0.02-0.04) | 0.04  (0.0-0.04) |  | 0.04  (0.0-0.04) | 0.04  (0.0-0.04) |  | 0.02  (0.0-0.04) | **0.0***  **(0.0-0.04)** |  | 0.0  (0.0-0.04) | 0.0  (0.0-0.04) |  | 0.0  (0.0-0.04) | 0.0  (0.0-0.04) |
| Serum Lactate |  | 2.1  (0.9-6.3) | 2.5  (0.7-4.6) |  | 2.2  (0.6-6.5) | 2.1  (0.9-4.2) |  | 1.9  (0.7-7.9) | 1.9  (0.8-4.0) |  | 1.6  (0.7-5.1) | 1.6  (0.7-4.7) |  | 1.5  (0.6-7.0) | 1.6  (0.9-13.2) |  | 1.3  (0.6-11.2) | 1.7  (0.6-9.1) |
| DO_2_I |  | 471  (239-1,028) | 415  (238-622) |  | 444  (186-1,069) | 473  (251-713) |  | 417  (173-955) | 455  (242-547) |  | 438  (210-925) | 402  (251-565) |  | 453  (311-896) | **395***  **(315-548)** |  | 459  (289-789) | 389  (289-663) |
| VO_2_I |  | 101  (41-242) | 93  (39-184) |  | 109  (47-230) | 97  (65-189) |  | 94  (28-189) | 100  (62-196) |  | 94  (37-197) | 97  (54-174) |  | 104  (57-181) | 98  (72-164) |  | 107  (58-173) | 102  (33-179) |
| O_2_ER x CO |  | 1,310  (553-3,212) | 1,265  (232-2,714) |  | 1,393  (645-4,100) | 1,372  (599-3,122) |  | 1,399  (781-3,546) | 1,537  (514-3,154) |  | 1,580  (396-2,387) | 1,294  (696-2,903) |  | 1,486  (681-2,499) | 1,463  (804-3,050) |  | 1,655  (584-3,026) | 1,491  (430-3,561) |

Supplementary Table 2. Hemodynamic & Vasopressor Drugs Monitoring in MB and Controls

*Data are express in median values (minimum and maximum). MB=Methylene Blue group; MAP=Median Arterial Pressure (mmHg); Norepinephrine (μg/kg/min); Vasopressin (IU/min); Serum Lactate (mmol/L); DO_2_I = Oxygen Delivery Index (mL/min/m^2^); VO_2_I = Oxygen Consumption Index (mL/min/m^2^); O_2_ER = Oxygen Extraction Rate (% x L/min); CO = Cardiac Output (L/min). Comparative analysis between Control x MB groups were assessed by Mann-Whitney test and significant differences at p<0.05 are highlighted by bold format and *.
