# Supplementary material for: Methylene blue therapy in addition to standard treatment for acute-phase septic shock: a pilot randomized controlled trial
Source: Front Med (Lausanne). 2024 Oct 14;11:1431321. doi: 10.3389/fmed.2024.1431321 (PMC11514138; doi:10.3389/fmed.2024.1431321)
Supplement: Supplementary file 5 [file Table_3.DOCX]

| Parameters |  | Kinetics Timeline | | | | | | | | | | | | | | | | |
| --- | --- | --- | --- | --- | --- | --- | --- | --- | --- | --- | --- | --- | --- | --- | --- | --- | --- | --- |
|  |  | T1 | |  | T2 | |  | T3 | |  | T4 | |  | T5 | |  | T6 | |
|  |  | Control | MB |  | Control | MB |  | Control | MB |  | Control | MB |  | Control | MB |  | Control | MB |
| CXCL8 |  | 318.0  (117-418) | 284.0  (120-478) |  | 257.5  (149-387) | 271.4  (127-491) |  | 247.1  (105-416) | 319.2  (127-553) |  | 244.4  (110-573) | 273.1  (156-385) |  | 226.3  (75-471) | 222.4  (188-419) |  | 277.4  (114-475) | 345.0  (228-454) |
| IL-6 |  | 425.3  (224-916) | 311.1  (197-1,037) |  | 447.8  (195-745) | 406.7  (204-1,238) |  | 409.6  (217-652) | 345.1  (227-1,054) |  | 192.6  (81-329) | 168.3  (78-573) |  | 108.2  (37-239) | 137.6  (77-427) |  | 149.2  (53-306) | 176.4  (95-229) |
| TNF-α |  | 17.1  (4.8-43) | 8.9  (6.0-24) |  | 17.7  (4.6-31) | 9.2  (4.4-41) |  | 18.8  (7.1-30) | 7.4  (5.0- 38) |  | 19.0  (14-29) | 12.0  (7.5-21) |  | 25.7  (9.4-51) | **8.8***  **(4.7-17)** |  | 24.5  (8.4-56) | **9.6***  **(6.0-18)** |
| IL-10 |  | 43.4  (32-86) | **88.4***  **(43-107)** |  | 40.2  (25-77) | 66.3  (44-82) |  | 44.3  (26-86) | 62.4  (44-110) |  | 41.0  (20-101) | 49.6  (35-67) |  | 47.1  (19-114) | 68.7  (41-85) |  | 47.2  (27-109) | **70.9***  **(56-959)** |
| NO |  | 70.0  (40-102) | 71.5  (39-128) |  | 67.7  (39-108) | 73.4  (41-140) |  | 68.6  (46-102) | 72.6  (40-148) |  | 44.7  (23-94) | **77.6***  **(52-126)** |  | 36.2  (20-72) | **67.6***  **(42-112)** |  | 32.1  (23-57) | 50.7  (29-90) |

Supplementary Table 3. Profile of plasmatic immune mediators and nitric oxide in MB and Controls

Data are presented in median values (interquartile range), expressed in pg/mL (CXCL8, IL-6, TNF-α and IL-10) and in μM (NO). Comparative analysis between Control x MB groups were assessed by Mann-Whitney test and significant differences at p< 0.05 highlighted by bold format and *.
